# Postmortem Evidence of CRH Neuron Reduction in Narcolepsy Without Cataplexy With Borderline Hypocretin‐1 Levels

**DOI:** 10.1111/jsr.70395

**Published:** 2026-06-25

**Authors:** Ling Shan, Suzan Linssen, Rolf Fronczek, Lucie Barateau, Christelle Peyron, Yves Dauvilliers

**Affiliations:** ^1^ Department Neuropsychiatric Disorders, Netherlands Institute for Neuroscience An Institute of the Royal Netherlands Academy of Arts and Sciences Amsterdam the Netherlands; ^2^ Department of Neurology Leiden University Medical Centre Leiden the Netherlands; ^3^ Sleep Wake Centre SEIN Heemstede the Netherlands; ^4^ Stichting Epilepsie Instellingen Nederland (SEIN) Sleep‐Wake Centre Heemstede the Netherlands; ^5^ Sleep‐Wake Disorders Unit, Department of Neurology Gui‐de‐Chauliac Hospital, CHU Montpellier France; ^6^ National Reference Centre for Orphan Diseases, Narcolepsy, Idiopathic Hypersomnia, and Kleine‐Levin Syndrome Montpellier France; ^7^ Institute of Neurosciences of Montpellier University of Montpellier, INSERM Montpellier France; ^8^ Centre de Recherche en Neurosciences de Lyon, CNRS UMR5292, Inserm U1028 Université Claude‐Bernard‐Lyon1 Bron France

**Keywords:** corticotropin‐releasing hormone, hypocretin (orexin), narcolepsy without cataplexy

## Abstract

Narcolepsy is classified as type 1 (NT1) or type 2 (NT2) mainly according to hypocretin deficiency rather than cataplexy. While CRH neuron loss in the PVN has been described in NT1, it remains unclear whether similar changes occur in narcolepsy without cataplexy. We report a 42‐year‐old woman with excessive daytime sleepiness since age 15, without cataplexy. Two multiple sleep latency tests and lumbar punctures performed 8 years apart showed mean latencies of 8.3 min with three SOREMPs and 10.8 min with two SOREMPs, along with borderline low‐intermediate hypocretin‐1 levels of 100 pg/mL initially and 122 pg/mL at follow‐up. HLA DQB1*06:02 was positive, and brain magnetic resonance imaging was normal. The patient later developed severe depression and committed suicide. CRH neuron counts were compared with a control and a NT1 hypothalamus. All hypothalami were formalin‐fixed, paraffin‐embedded and processed for thionin staining and immunohistochemistry for hypocretin and CRH, with neurons manually counted. Only the anterior hypothalamus was available from this case, precluding the assessment of hypocretin neurons, although CSF levels indicated a borderline low‐intermediate reduction. Thionin staining confirmed that the PVN was fully included in our case and revealed an almost complete loss of CRH‐positive neurons in the PVN compared with controls, consistent with findings in NT1. This report indicates that narcolepsy without cataplexy, accompanied by borderline low CSF hypocretin‐1 levels, is associated with a marked reduction in CRH neurons in the PVN, which may contribute to excessive daytime sleepiness and impaired wakefulness regulation, without triggering cataplexy.

## 1 Introduction

Narcolepsy is currently classified into narcolepsy type 1 (NT1) and narcolepsy type 2 (NT2), rather than narcolepsy with or without cataplexy (Bassetti et al. 2019). This distinction is primarily based on the presence of hypocretin (orexin) deficiency in NT1. However, some patients without cataplexy exhibit low cerebrospinal fluid (CSF) hypocretin‐1 levels, while some patients with cataplexy have normal values, resulting in incompletely defined diagnostic boundaries (Khatami et al. 2004; Andlauer et al. 2012; Postiglione et al. 2022). Hypocretin neurons are almost completely lost in NT1 (Peyron et al. 2000; Thannickal et al. 2000), whereas the few available postmortem studies suggest a partial loss in NT2 (Thannickal et al. 2009), although their rarity limits firm conclusions. As a result, the pathophysiology of NT2 remains poorly understood and highly heterogeneous. Previous work has demonstrated an 88% reduction of corticotropin‐releasing hormone (CRH) neurons in the paraventricular nucleus (PVN) in NT1 (Shan et al. 2023). However, it is currently unknown whether CRH neurons are similarly affected in narcolepsy without cataplexy.

Here, we report the clinical and neuropathological findings of a patient with narcolepsy without cataplexy and borderline low CSF hypocretin‐1 levels (measured twice; 100 pg/mL initially and 122 pg/mL at follow‐up), and compare hypothalamic CRH neuron counts with those of an individual with typical NT1 and a control subject.

### 1.1 Report of a Case

A 42‐year‐old woman presented with excessive daytime sleepiness since age 15, without cataplexy or family history of sleep disorders. She reported frequent refreshing sleep episodes, hypnagogic hallucinations and recurrent sleep paralysis. Epworth Sleepiness Scale score was 21, and BMI was 24.0 kg/m^2^. Night‐time sleep was generally restorative, with a habitual schedule of 23:00–07:00 on weekdays and prolonged sleep until 10:00 during weekends, accompanied by mild sleep inertia.

Baseline polysomnography in 2004 showed good sleep efficiency (90%), REM latency of 39 min, low apnea–hypopnea index (1.9/h), and minimal periodic limb movements (2.4/h). Multiple sleep latency test revealed a mean sleep latency of 8.3 min with three sleep‐onset REM periods (SOREMPs). Brain MRI was normal. CSF hypocretin‐1 level was 100 pg/mL (radioimmunoassay), and HLA DQB1*06:02 was positive. Modafinil was initiated. Repeat PSG/MSLT in 2012, following modafinil washout, showed a mean sleep latency of 10.8 min with two SOREMPs. A second lumbar puncture demonstrated a CSF hypocretin‐1 level of 122 pg/mL. Pitolisant was initiated but discontinued due to insomnia; treatment with methylphenidate was effective. Cataplexy remained absent. In 2017, the patient developed severe depression and anxiety and died by suicide the following year. Postmortem examination revealed no macroscopic abnormalities.

Brain tissue (18ER0023) was preserved for subsequent scientific investigation; the study was approved by the local institutional review board. Two additional hypothalami were obtained from the Netherlands Brain Bank: a typical NT1 case (NBB2008‐023; previously reported (Shan et al. 2022), female, 66 years old; HLA DQB1*06:02 positivity; cause of death: heart failure; relevant medication: amphetamine, modafinil, oxybate, undetectable level of CSF hypocretin‐1) and a female control (NBB1999‐125, 40 years, cause of death: sepsis with multi‐organ failure; relevant medication: methylprednisolone). The control was selected with the same sex and similar age rank of the case of 18ER0023. The hypothalami of the subjects were formalin‐fixed, embedded in paraffin, and serially sectioned at 6 μm intervals along the rostro‐caudal axis. Every 100th section was stained with thionin to determine the borders of the PVN and medial‐lateral hypothalamus for hypocretin region.

#### 1.1.1 Immunocytochemistry Hypocretin and CRH

The hypocretin and CRH staining were performed according to previous published procedures (Shan et al. 2022, 2023). In short for hypocretin immunocytochemistry, antigen retrieval was performed: sections were microwaved for 10 min in the 0.01 M sodium citrate buffer (pH 6.0). After washing the slides in Tris‐buffered saline (TBS), the sections were pre‐treated in TBS‐0.5% milk for 1 h at room temperature and incubated with the primary antibody rabbit IgG, anti‐hypocretin‐1 1:20.000 diluted in Supermix (SUMI) (TBS 0.25% gelatin, 0.5% Triton X‐100)‐0.5% milk for 1 h, at room temperature, followed by overnight incubation at 4°C in a moist chamber. After the primary antibody incubation, the slides were rinsed in TBS‐0.5% milk and washed in TBS before they were incubated with the secondary antibody (biotinylated horse anti‐rabbit IgG 1:400, in SUMI) at room temperature for 1 h. The slides were thoroughly washed in TBS and incubated in a 1:800 SUMI ABC complex for 1 h at room temperature. Thereafter, the slides were rinsed in TBS and incubated with the DAB Nickel solution for 20 min, after which the reaction was stopped in distilled water. Finally, the slides were dehydrated in the graded ethanol and cleared in Xylene. CRH immunocytochemistry staining was carried out as described above for hypocretin, with the exceptions that no antigen retrieval or pre‐incubation steps were needed. Rat IgG, PFU83 (1:100.000) was used as primary antibody. As a secondary antibody, we used biotinylated anti‐rat IgG 1:400 in SUMI (Shan et al. 2023).

#### 1.1.2 Quantification Strategies

Images of the hypocretin and CRH stained sections were acquired by scanning the sections in the tissue scanner Zeiss Axioskop Plan‐NEOFLUAR Zeiss objectives (Carl Zeiss GmbH, Jena, Germany). The areas of interest were manually outlined on the computer after scanning at a ×2.5 objective magnification. Thereafter, ×40 objective images of the outlined areas were generated. These high‐resolution images were uploaded to QuPath version 0.2.3 software and used to manually count neuronal cell numbers. To prevent double counting, only neurons with a visible nucleolus were counted.

## 2 Results

In the control subject, hypocretin cell counts peaked between the region where the fornix passes the PVN and where it reaches the mammillary bodies (Aziz et al. 2008), while CRH cell counts peaked at the level of the PVN (Figure 1). In case 18ER0023‐A2a, the PVN spanned sections 500–1500. The hypocretin region was not available for analysis, as the tissue section ended as the fornix was passing through the PVN (Figure 1). In contrast, both the control case NBB 1999‐125 (PVN: 500–1400; hypocretin: 600–1800) and the NT1 case NBB2008‐023 (PVN: 750–1300; hypocretin: 1000–1700) showed a complete PVN and an intact hypocretin region extending to the level where the mammillary body appeared. 18ER0023‐A2a hypothalamic sections exhibited an almost complete loss of CRH‐positive neurons compared with the matched control, consistent with the CRH neuron loss observed in the NT1 case (Figure 1B). However, it should be noted that the hypocretin region of this case was completely missing, which prevents us from quantifying hypocretin neurons.

**FIGURE 1:**
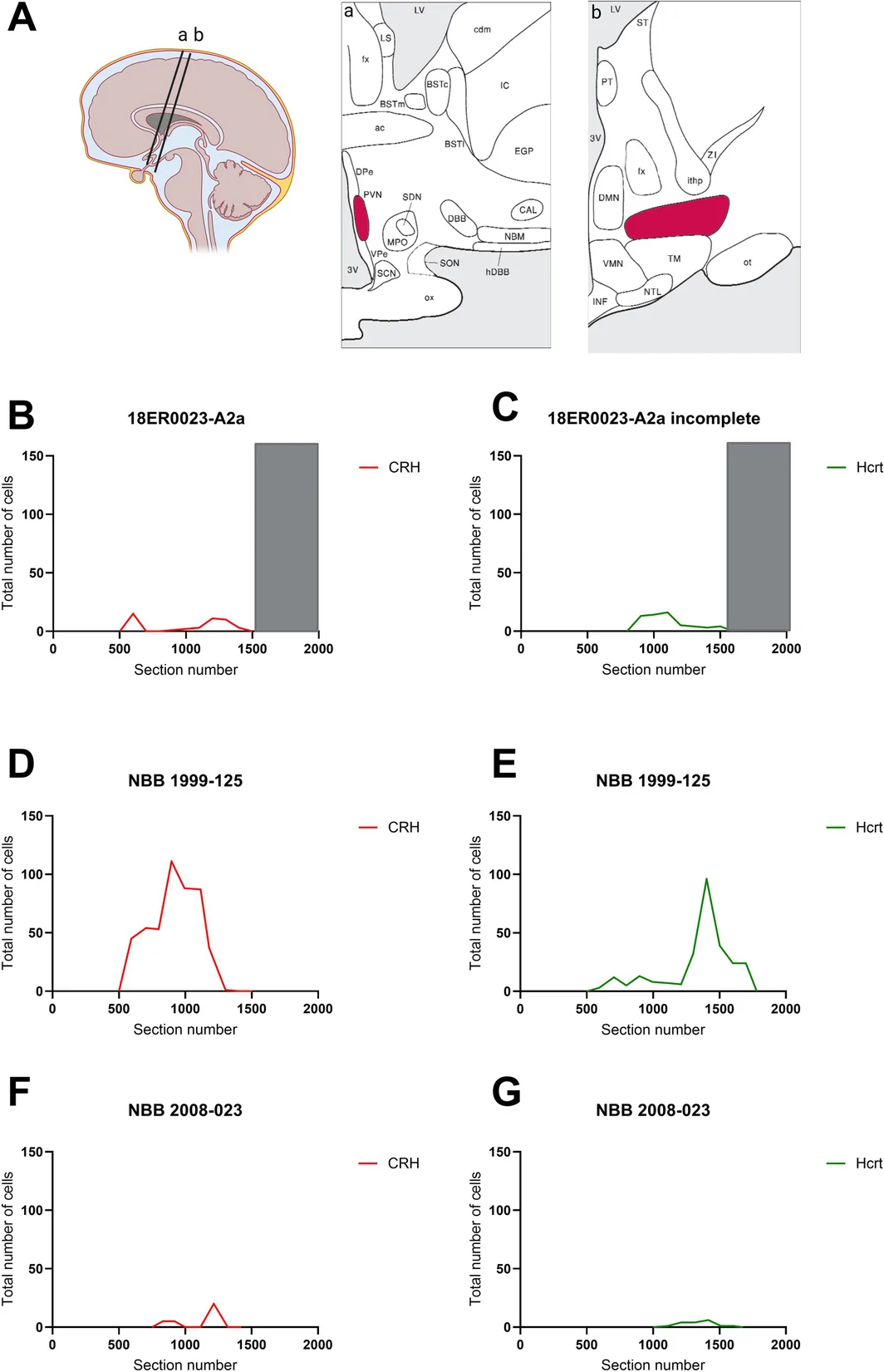
The loss of Corticotropin‐releasing hormone (CRH) cell in the hypothalamus of narcolepsy without cataplexy patient that missed the hypocretin region. (A) Schematic illustration of the anterior and medial hypothalamus. The borders were further confirmed by immunohistochemically staining CRH and hypocretin neurons. The dorsal boundary of the PVN was set at the hypothalamic sulcus, corresponding to the border between thalamus and hypothalamus (Koutcherov et al. 2000; Swaab 2003). (a) The anterior PVN was defined at the first level where the third ventricle becomes clearly visible and the columns of the fornix begin to descend in proximity to the anterior commissure (Koutcherov et al. 2000; Swaab 2003). (b) The posterior PVN was defined as the last level at which PVN neurons were present before the descending columns of the fornix had passed through the region (Koutcherov et al. 2000; Swaab 2003). This posterior level coincided with the rostral plane where hypocretin are just appear in the medial–lateral hypothalamus. The hypocretin neurons stopped when the mammillary bodies are visible (Koutcherov et al. 2000; Swaab 2003). (B) Narcolepsy without cataplexy patient 18ER0023‐A2a. Few cells are CRH positive. (C) Hypocretin (HCRT) neuronal distribution in the same patient and areas without available tissue are indicated by the grey boxes. Due to limited tissue availability, the hypothalamic sections did not encompass the full rostro caudal distribution of HCRT neurons. HCRT (D) and CRH (E) neuronal distribution in Control (NBB 1999‐125), total number of cell levels are covered. Narcolepsy type 1 (NT1) patient NBB 2008‐023, few neurons are CRH (F) or HCRT (G) positive, but the whole regions were covered.

## 3 Discussion

We report the first observation of markedly reduced CRH neurons in the PVN of a patient with well‐characterized narcolepsy without cataplexy and borderline low‐intermediate CSF hypocretin‐1 levels, measured twice over an eight‐year interval, compared with a matched control. This finding is consistent with CRH neuron loss observed in NT1. Only the anterior hypothalamus was available for analysis, preventing direct assessment of hypocretin neurons; however, CSF hypocretin‐1 levels, which correlate with hypocretin cell numbers, indicate a partial reduction in this case.

Distinguishing NT1 from NT2 can remain challenging (Baumann et al. 2014; Lammers et al. 2020; ICSD 2023). The patient exhibited typical narcoleptic symptoms, including excessive daytime sleepiness with short, refreshing naps often associated with dreaming, hypnagogic hallucinations and recurrent sleep paralysis, along with HLA DQB1*06:02 positivity but no cataplexy. MSLTs performed twice off CNS‐active medications showed multiple SOREMPs, although mean sleep latencies were above the pathological threshold of 8 min on both occasions. CSF hypocretin‐1 was initially 100 pg/mL, below the conventional 110 pg/mL cutoff favouring NT1, while a repeat lumbar puncture measured 122 pg/mL, consistent with partial hypocretin loss (ICSD 2023). In addition, variability between radioimmunoassay is substantial, and alternative quantification methods, such as mass spectrometry, are under development (Vialaret et al. 2005). Milder or partial forms of narcolepsy characterized by intermediate hypocretin levels have been described; however, it remains uncertain whether dysfunction of the hypocretinergic system alone can account for all narcolepsy related symptoms (Khatami et al. 2004; Postiglione et al. 2022).

The pathophysiology of narcolepsy without cataplexy is unknown and may be highly heterogeneous; however, a subset of patients with persistent symptoms and HLA DQB1*06:02 positivity may exhibit partial loss of hypocretin neurons, suggesting a dose‐dependent or threshold effect (Baumann et al. 2014; Lammers et al. 2020). Our findings revealed a marked reduction in CRH‐expressing neurons in the PVN, comparable to that observed in NT1 (Shan et al. 2023), supporting a shared, and potentially overlapping, pathophysiology between narcolepsy with and without cataplexy. Previous studies in mice have shown that optogenetic activation of CRH neurons in the PVN promotes wakefulness, whereas chemogenetic inhibition and DTA‐mediated ablation reduce wakefulness (Li et al. 2020; Ono et al. 2020) and that optogenetic modulation of CRH release in the thalamic reticular nucleus alters NREM sleep fragmentation (Cumpana et al. 2025). In our case, this pronounced reduction in CRH neurons was not associated with cataplexy but may contribute to excessive daytime sleepiness and may be involved in disrupted wakefulness regulation.

A key limitation of this study is the inclusion of only a single patient with narcolepsy without cataplexy as well as the incomplete hypothalamic tissue available for analysis; however, postmortem brains from such individuals are exceedingly rare. For this case, we provide detailed clinical characterization, with two PSG–MSLT evaluations and two CSF hypocretin‐1 assessments with borderline low‐intermediate levels.

In conclusion, although limited, these findings suggest that narcolepsy without cataplexy, accompanied by borderline low CSF hypocretin‐1 levels, is associated with a marked reduction in CRH neurons in the PVN, which may contribute to excessive daytime sleepiness and impaired wakefulness regulation without triggering cataplexy.

## Author Contributions

**Ling Shan:** investigation, writing – original draft, conceptualization, methodology, formal analysis. **Yves Dauvilliers:** conceptualization, investigation, writing – original draft, writing – review and editing, validation, methodology. **Rolf Fronczek:** writing – review and editing. **Lucie Barateau:** writing – review and editing. **Suzan Linssen:** investigation, writing – review and editing. **Christelle Peyron:** writing – review and editing, methodology.

## Funding

L.S. was supported by funds of the Netherlands Institute for Neuroscience Foundation.

## Conflicts of Interest

The authors declare no conflicts of interest.

## Data Availability

The data that support the findings of this study are available on request from the corresponding author. The data are not publicly available due to privacy or ethical restrictions.
